# Non-classical MHC I-E negatively regulates macrophage activation and Th17 cell development in NOD mice

**DOI:** 10.1038/srep12941

**Published:** 2015-08-07

**Authors:** Chunhui Yang, Nining Guo, Jinhua Liu, Juhao Yang, Kai Zhu, Hui Xiao, Qibin Leng

**Affiliations:** 1 Key Laboratory of Molecular Virology and Immunology, Institut Pasteur of Shanghai, Chinese Academy of Sciences, 320 Yueyang Road, Shanghai, China

## Abstract

Transgenic expression of I-E molecules prevents diabetes in NOD mice. So far, the precise role of these non-classical MHC II molecules remains elusive. Here, we showed that transgenic expression of I-E^k^ alpha 16 molecule in NOD mice selectively reduced Th17 cells in the thymus and pancreatic draining lymph nodes. The reduction in Th17 cells was associated with both attenuated IL-6 production and decreased activation of macrophages. Mechanistically, transgenic expression of the I-E molecule diminished expression of intracellular classical MHC II molecule and led to impaired TLR4-mediated signaling. In contrast to classical MHC II molecule, this non-classical MHC II molecule negatively regulates the inflammatory responses of macrophages. Altogether, our study reveals a novel regulatory role of I-E molecules in modulating inflammatory immune responses.

Type I Diabetes (TID) is an autoimmune disease caused by the immune destruction of insulin-producing β cells in the pancreas^1^,^2^,^3^. Susceptibility to the disease is primarily associated with the major histocompatibility complex (MHC) in both humans and mice. Non-obese Diabetic (NOD) mice spontaneously develop TID and are frequently used for studying the etiology of this disease^1^,^2^,^3^. NOD mice not only express a distinct classical MHC II isoform, namely I-A^g7^, but also do not express non-classical MHC-II I-E molecules due to a deletion of the alpha chain gene^1^,^2^,^3^. The presence of a unique classical MHC-II molecule and the absence of the I-E molecule both contribute to the susceptibility of NOD mice to TID^2^,^3^.

The protective role of I-E molecules in the development of diabetes is supported by evidence showing that I-E transgenic mice have no or much lower incidence of diabetes than wild-type NOD mice^4^,^5^,^6^. The effect of transgenic I-E expression in NOD mice was initially linked to ontogenetic deletions of self-reactive CD4^+^ T cell clones during negative selection in the thymus. The hypothesis that the I-E molecule mediates the negative selection of self-reactive T cells is based on the observation that T cells harboring the TCRs Vβ5, Vβ6 and Vβ11 are significantly reduced in I-E transgenic mice^5^,^7^,^8^. However, later studies did not support the hypothesis that the clonal deletion of self-reactive T cells accounts for diabetes protection in NOD-E mice^5^,^6^,^9^,^10^. Therefore, the mechanism by which the I-E molecule protects NOD mice from diabetes remains elusive.

In addition to affecting the development and function of T cells, MHC II molecules have also been found to promote innate immune responses. Two early studies showed that cross-linking MHC II molecules with superantigen or monoclonal antibody triggers the production of inflammatory cytokines and nitric oxide by macrophages^11^,^12^. Consistent with this behavior, a defect in MHC class II expression has been shown to cause diminished secretion of pro-inflammatory cytokines by macrophages following stimulation with lipopolysaccharide (LPS)^13^. Recently, MHC II molecules have been shown to promote inflammatory cytokines expression by macrophages and dendritic cells by enhancing Toll-like receptor signaling through its intracellular interaction with BTK and CD40^14^. However, this mechanistic study was mainly based on H2-knockout mice that lack both classical and non-classical MHC II molecules. Therefore, it remains unknown whether non-classical MHC II molecules have the same or different regulatory roles in innate immune responses.

The discovery of a regulatory role for MHC II molecules in innate immunity led us to wonder whether the diabetes protective effect of the non-classical I-E molecule is due to direct effects on innate immune responses or/and indirect effects on adaptive immune responses in NOD mice. Therefore, we studied the effects of the I-E molecule on T cell development and innate immune responses. We found that transgenic I-E expression tended to decrease Th17 cell development in NOD mice. This decrease in Th17 cells was associated with decreased levels of IL-6 expression, as well as selectively diminished innate signaling in CD11b^+^ macrophages. Our study suggests that the I-E molecule, unlike classical MHC II molecules, plays a negatively role in innate immune responses.

## Results

### Reduced induction of Th17 cells in I-E transgenic mice

**Figure 1:**
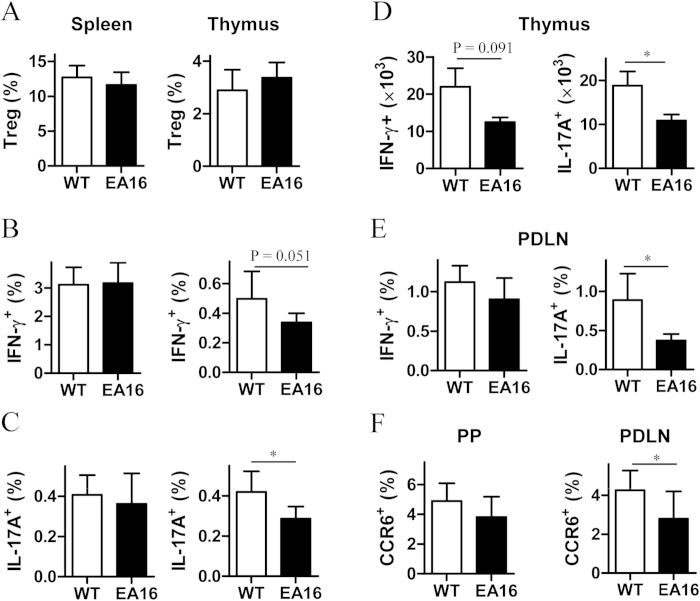
Effects of transgenic I-E expression on the development of CD4^+^ T cell subsets. (**A-C**) Percentages of CD25^+^Foxp3^+^ Treg (**A**), IFN-γ-producing Th1 (**B**) and IL-17A-producing Th17 (**C**) cell populations in CD4^+^CD8^-^ single positive (CD4 SP) cells from the spleens and thymi of Ea16-transgenic (EA16) and wild-type (WT) NOD mice. (**D**) Absolute numbers of Th1 and Th17 populations in the thymi from EA16 and WT mice were shown. (**E-F**) Percentages of the Th subsets (**E**) and CD44^+^CCR6^+^ population (**F**) in CD4^+^ T cells in PPs and PDLNs from EA16 and WT NOD mice were shown. Data (mean ± SEM) are representative of two (**E**) or three (**A-D,F**) independent experiments with similar results (n = 6 per group). *P < 0.05.

Given the contradictory findings regarding the role of transgenic I-E in the negative selection of self-reactive CD4^+^ T cells, we wondered whether the I-E molecule affects the development of CD4^+^ T-cell subsets in I-E transgenic mice. To test this possibility, we examined Th1, Th17 and regulatory T (Treg) subsets in wild-type (WT) NOD mice and in EA16 mice, which transgenically express the I-E^k^ alpha 16 gene^6^. The percentages of Treg cells in the thymus and spleen of EA16 mice did not differ significantly from those of WT NOD mice (Fig. 1A). Similarly, the percentages of IFN-γ-producing (Th1) and IL-17A-producing (Th17) CD4^+^ T cells in the spleen of EA16 mice were not significantly different from those of WT NOD mice (Fig. 1B-C); whereas in the thymus, Th1 and Th17 cells of EA16 mice dropped proximately 68% and 56%, respectively (Fig. 1B-D). We further examined the proliferation rates of thymocytes with intracellular staining of Ki-67, a proliferation marker. Although both Th1 an Th17 cells in thymus similarly proliferated 4–5 times higher than total CD4 single-positive thymocytes, the proliferation rates of these two subsets in EA16 mice were not significantly different from WT NOD mice (Fig. S1). Thus, the decease of thymic Th1 and Th17 cells of EA16 mice unlikely results from a decrease of proliferation.

Th17 cells preferentially express CCR6 and can be recruited by its ligand, CCL20^15^,^16^. We thus examined CCL20 expression in the thymi of EA16 and WT NOD mice with quantitative reverse transcription polymerase chain reaction (qRT-PCR). Our results revealed that the CCL20 expression levels in EA16 mice were not significantly different from those in NOD WT mice (Fig. S2). Thus, the decrease of Th17 cells in EA16 mice is also unlikely due to a decline of CCL20-mediated migration of Th17 cells into thymus.

We also examined Th1 and Th17 cells in pancreatic draining lymph nodes (PDLNs), which are directly involved in the development of diabetes. Although the proportion of Th1 cells in the PDLNs of EA16 mice was not significantly different from WT NOD mice, the percentage of Th17 cells in the PDLNs of EA16 mice was approximately 60% lower (Fig. 1E). A similar decline in the absolute numbers (18.2 ± 3.6 × 10^3^ Th17 cells versus 7.5 ± 0.9 × 10^3^ Th17 cells) were also observed. Peyer’s patch (PP) is also an induction site for Th17 cells^17^,^18^. Therefore, we examined the CCR6^+^CD4^+^ T cells in PDLNs and PPs. We observed a significant decrease in CCR6^+^CD44^+^CD4^+^ T cells in PDLNs of EA16 mice compared with WT NOD mice, whereas the proportion of CCR6^+^CD44^+^CD4^+^ T cells in PP in EA16 and WT NOD mice did not significantly differ (Fig. 1F). In addition, the decrease of CCR6^+^CD44^+^CD4^+^ T cells in PDLNs of EA16 mice was not related to the proliferation rates of these cells (Fig. S1). Taken together, our results suggest that the transgenic expression of the I-E molecule preferentially induces organ-specific reductions in Th17 cells.

### Th17 cell reduction is not associated with specific depletion of T-cell clones mediated by I-E molecule

**Figure 2:**
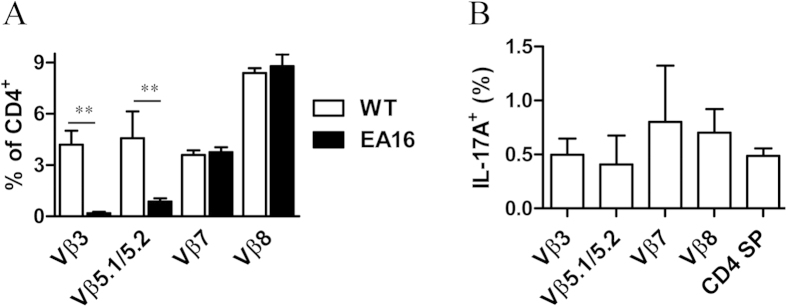
Effects of transgenic I-E expression on the TCR Vβ usage of CD4 SP thymocytes and on TCR Vβ-specific Th17 subsets. (**A**) The frequency of cells positive for each indicated TCR Vβ chain among CD4^+^ SP thymocytes from EA16 and WT NOD mice was analyzed by flow cytometry. (**B**) Percentages of Th17 cells in indicated TCR Vβ-positive CD4^+^ SP thymocytes or in total CD4^+^ SP thymocytes. Data (mean ± SEM) are representative of two independent experiments with similar results (n = 6 per genotype). **, *P* < 0.01 .

Different I-E molecules have been linked with the preferential depletion of several specific T-cell clones with different TCR Vβ chains^7^,^8^,^19^,^20^. To examine the influence of I-E^k^ alpha on TCR Vβ chain usage in EA16 mouse thymocytes, we compared the TCR Vβ chain usage preference of EA16 and NOD mice. In keeping with previous findings^6^,^19^, the frequency of CD4^+^ single-positive (SP) thymocytes with TCR Vβ5 was dramatically reduced in EA16 mice. Similarly, the frequency of CD4^+^ SP thymocytes with TCR Vβ3 was also significantly decreased in EA16 mice, suggesting that I-E^k^ alpha expression could also cause the deletion of the TCR Vβ3 thymocyte fraction (Fig. 2A). The deletion of thymocytes with Vβ3 and Vβ5 TCRs appeared to be a specific effect of I-E^k^ alpha because Vβ8.1/8.2/8.3 and Vβ13 thymocytes seemed not to be affected by transgenic I-E expression in EA16 mice (Fig. 2A).

The recognition of self antigens promotes the development of natural Th17^+^ cells in the thymus^21^. If thymocytes with Vβ3 and Vβ5 TCRs are self-reactive, they would preferentially differentiate into Th17 cells. We therefore examined if there was any preference for the usage of TCR Vβs by Th17 thymocytes in NOD mice. In contrast to the TCR Vβ usage in total CD4^+^ SP thymocytes, the proportion of IL-17A^+^ CD4^+^ SP thymocytes among all tested TCR Vβ chains was not significantly different (Fig. 2B). In other words, the proportional reduction of Th17 cells in EA16 mice was not likely the result of the preferential deletion of these TCR Vβ subsets.

### Transgenic I-E attenuates IL-6 expression and macrophage activation

**Figure 3:**
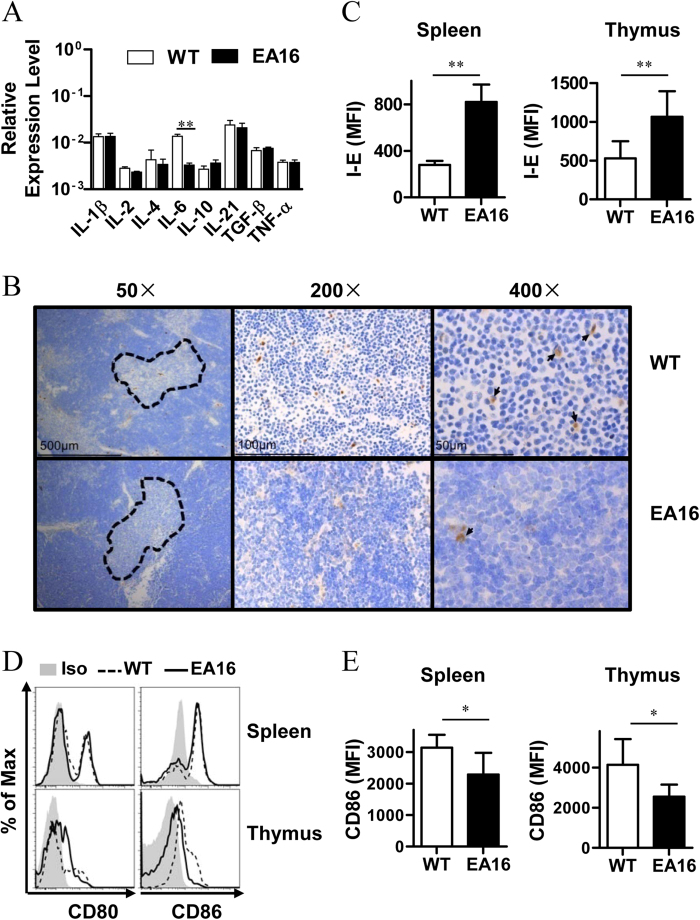
I-E molecule attenuates macrophage activation and proinflammatory cytokine production. (**A**) qRT-PCR analysis of the cytokine profile in the thymi from EA16 and WT NOD mice. Expression levels of cytokines were normalized to GAPDH. (**B**) Representative images (n = 5) for the histological analysis of thymus tissue from EA16 (below) and WT (above) NOD mice. The samples were processed for both immunohistochemical staining with anti-IL-6 antibody and haematoxylin counterstaining to detect IL-6 producing cells. The medulla areas that had high staining of IL-6 were further magnified. (**C**) Mean fluorescence intensity (MFI) of transgenic I-E expression on CD11b^+^ macrophages in the spleens and thymi from EA16 and WT NOD mice. CD11b^+^ macrophages were gated on the CD3e^-^B220^-^ CD11b^+^ population. (**D**) CD80 and CD86 expression levels on CD11b^+^ macrophages in the spleens and thymi from EA16 (solid line) and WT (dashed line) NOD mice were detected by flow cytometry. (**E**) MFI of CD86 expression from (**D**). Data (mean ± SEM) are representative of two independent experiments (**A,B**) (n = 6 per genotype) or three independent experiments (**C–E**) with similar results (n = 3 per genotype). *P < 0.05.

To characterize the cytokine milieu that is associated with the decreased level of Th17 cells in EA16 mice, we examined the expression levels of cytokines that are crucial for Th17 development by quantitative reverse transcription-PCR (qRT-PCR). We found that the expression of cytokines, including IL-1β, IL-2, IL-4, IL-10, IL-21, TGF-β and TNF-α, in the thymus of EA16 mice was not significantly different from that of WT NOD mice (Fig. 3A and Fig. S3). In contrast, IL-6 expression levels in EA16 mice dropped to 22% of that in WT NOD mice (Fig. 3A). ELISA assay and immunohistochemical (IHC) staining also confirmed that EA16 mice indeed had less IL-6 expression in thymus than WT NOD mice (Fig. 3B and Fig. S3).

CD11b^+^ cells (mainly macrophages) have been reported to play a critical role in Th17 differentiation in NOD mice^22^,^23^, and we observed transgenic I-E expression in the macrophages of EA16 mice (Fig. 3C). In addition, the IL-6 expression levels of CD11b-depleted thymocytes from EA16 mice were not significantly (p = 0.834) different from those from WT NOD mice (Fig. S4), implying that macrophages may mainly contribute to the difference of IL-6 expression. Therefore, we also examined the effect of I-E^k^ alpha expression on macrophage activation in the spleen and thymus of EA16 mice. We found that macrophages from EA16 mice expressed a comparable level of CD80 as those from WT NOD mice (Fig. 3D). Notably, these cells had significantly lower levels of CD86 expression in EA16 mice (Fig. 3D,E), which indicates the attenuation of macrophage activation by I-E^k^ alpha.

### Selective attenuation of macrophage signaling is linked to the downregulation of intracellular I-A expression by transgenic I-E expression

**Figure 4:**
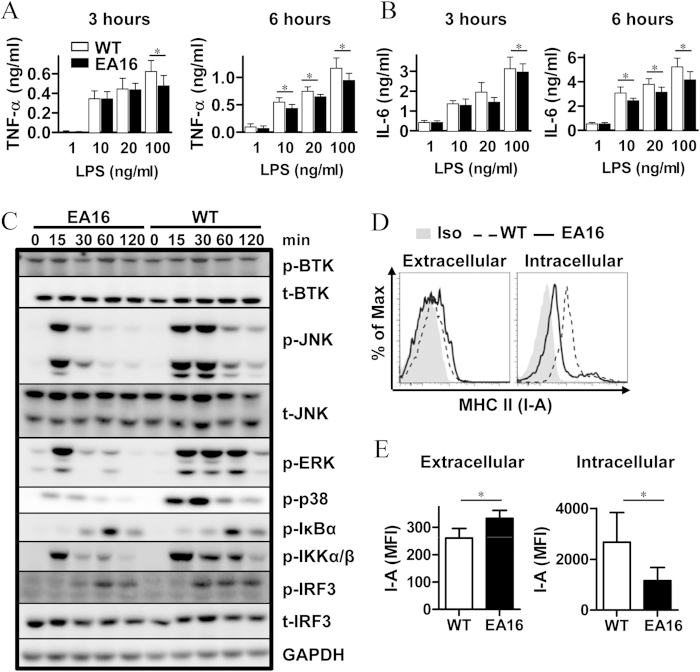
The molecular mechanisms of I-E-attenuated macrophage activation. (**A,B**) Bone-marrow-derived macrophages (BMMs) from EA16 and WT NOD mice were stimulated with the indicated concentration of LPS for 3 or 6 h, and the levels of TNF-α and IL-6 in the supernatants were determined by ELISA. (**C**) Immunoblot of phosphorylated (p-) or total (t-) proteins in lysates of WT or EA16 BMMs stimulated with LPS (100 ng/ml) for the indicated times. (**D**) Flow cytometry analysis of the extracellular or intracellular expression of I-A by BMMs of EA16 or WT NOD mice. Isotype-matched antibody (Iso) was used as a negative control for I-A staining. (**E**) MFI of extracellular or intracellular I-A molecule expression by BMMs of EA16 or WT NOD mice. Data (mean ± SEM) are representative of three independent experiments with similar results (**C**) or are from two independent experiments (**A–E**, n = 6 per genotype). *P < 0.05; **,

To corroborate these *in vivo* findings, we also examined the production of IL-6 and TNFα by bone marrow-derived macrophages (BMMs), which were differentiated *in vitro* from the bone marrow of EA16 mice and WT NOD mice. Upon stimulation with a high concentration of the Toll-like receptor 4 (TLR4) ligand LPS, EA16 BMMs produced significantly less TNF-α and IL-6 than WT BMMs (Fig. 4A,B). This observation suggests that transgenic I-E intrinsically suppresses the activation of macrophages.

Next, we sought to elucidate the molecular mechanism underlying the I-E-mediated attenuation of macrophage activation. We first investigated the effect of I-E expression on TLR4-activated downstream signaling pathways in macrophages. We did not observe any defect in BTK activation in EA16 BMMs. In contrast, the phosphorylation of ERK1/2, JNK, p38 MAPK, IKKα/β and IκB was impaired in LPS-stimulated EA16 BMMs (Fig. 4C). We’ve also examined the effect of I-E expression on the signaling triggered by zymosan, a stimulus for TLR2 and dectin-1. A similar impaired signaling was observed (Fig. S5). In addition, the downstream activation of IRF3 was attenuated in LPS-treated EA16 BMMs (Fig. 4C). Thus, the attenuation of macrophage activation by I-E is related to defects in the activation of the MAPK and NF-κB pathways.

Intracellular MHC class II molecules have been shown to promote TLR signaling in macrophages. We therefore wondered whether I-E, as a non-classical MHC II molecule, may interfere with the expression of classical MHC II. We next examined both the intracellular and extracellular levels of I-A expression in EA16 and WT NOD mice. The extracellular expression of I-A on EA16 BMMs was slightly but significantly increased by an average of approximately 21%. In contrast, intracellular I-A levels were down-regulated in EA16 BMMs by an average of approximately 60% (Fig. 4D,E). Hence, the downregulation of intracellular MHC class II expression may be associated with defects in TLR signaling.

## Discussion

Transgenic expression of I-E molecule, a nonclassical MHC II molecule, protects NOD mice from diabetes. So far, the regulatory role of I-E molecule in immune responses remains elusive. In the present study, the transgenic expression of MHC class II I-E molecule was found to be related to a reduction of Th17 cells in the thymi and in pancreatic draining lymph nodes. Th17 cells are pathogenic in diabetes development^23^,^24^; thus, the reduction of Th17 cells may contribute to the protective role of I-E molecules in diabetes. Furthermore, although I-E expression did lead to the negative selection of TCR Vβ3 and Vβ5 T-cell clones, the decreased natural Th17 differentiation was unlikely to be associated with this negative selection. The reduced differentiation of natural Th17 cells in the thymus was instead associated with decreased IL-6 expression in macrophages. Further investigation revealed that the transgenic expression of I-E led to a decline in the intracellular expression of classical MHC II, which was linked to the attenuated signaling in macrophages. Consistent with these observations, I-E expression was previously reported to be inversely correlated with I-A expression and the development of TID^25^. Taken together, our study suggests that the suppression of I-E molecule in inflammatory immune response in NOD mice may result from down-regulation of classic MHC-II molecules.

In our present study, the I-E molecule was revealed to negatively regulate TLR signaling in macrophages. Paradoxically, Liu X *et al.* found that intracellular MHC class II molecules are required for TLR signaling to trigger the full activation of macrophages and dendritic cells. Technically, however, their observations were based on H-2 knockout mice, which lack both classical and non-classical MHC II molecules. Our results showed that the presence of the I-E molecule diminished the intracellular expression of classical MHC II molecule. Interestingly, I-E transgenic expression also slightly enhanced extracellular expression of classical MHC II molecule. Because the intracellular down-regulation was more dramatic than the extracellular up-regulation, it is unlikely that I-E molecule simply directly changes the localization of classical MHC II molecule. The underlying mechanism merits further to be fully investigated. Nevertheless, this finding emphasizes the idea proposed by Liu X *et al.*^14^ that intracellular MHC II molecules functionally are different from the extracellular ones.

The suppression of Th17 induction was observed only in the thymus and in pancreatic draining lymph nodes but not in the spleen or in Peyer’s patches. I-E expression levels in antigen-presenting cells have been shown to correlate with their suppressive function in the development of diabetes^25^. Therefore, I-E expression levels in macrophages and/or other antigen-presenting cells in these tissues may differ from those in the other tissues, thereby having different effects on Th17 induction.

Our present study suggested that the downregulation of classical MHC II expression by the I-E molecule may contribute to the inhibition of macrophage activation in response to LPS. Similarly, the reduced expression of the classical MHC II molecule HLA-DR on the peripheral blood monocytes of patients with septic shock was associated with the diminished response of these cells to LPS^26^,^27^. In addition, the restoration of HLA-DR expression in these patients via IFN-γ treatment substantially enhances the response of monocyte to LPS *in vitro*^28^. Thus, the expression levels of classical MHC II molecules may also be an important factor in the development of inflammatory diseases in humans, including type I diabetes. In particular, genetic polymorphisms in both the classical MHC II HLA DP and DR genes are tightly linked to the risk of type I diabetes in humans^1^,^29^,^30^. Thus, it will be interesting to investigate whether and how gene polymorphisms affect the expression levels of these classical MHC II molecules in antigen-presenting cells, as well as their regulatory functions in innate immune responses.

## Materials and Methods

### Mice and ethics statement

NOD/ShiLtJ mice were purchased from the Shanghai Laboratory Animal Center (SLAC). Eα16-NOD mice (cited as EA16) were a gift from Dr. Christopher Benoist. All mice were maintained in a specific pathogen-free (SPF) room at Institut Pasteur of Shanghai. All animal experiments were carried out in strict accordance with the regulations in the Guide for the Care and Use of Laboratory Animals issued by the Ministry of Science and Technology of the People’s Republic of China. All efforts were made to minimize suffering. The protocol was approved by the Institutional Animal Care and Use Committee of the Institut Pasteur of Shanghai (Permit Number: A2011005).

### Flow cytometry and antibodies

Cells were prepared in FACS staining buffer (PBS with 0.3% BSA and 0.1% sodium azide) and blocked with anti-CD16/CD32 (CL93, eBioscience, San Diego, CA) for 10 min on ice, followed by staining with fluorophore-conjugated antibodies. Cells were washed twice with FACS staining buffer, and data were collected on a FACS LSR II or Fortessa flow cytometer (BD Biosciences, San Diego, CA). For intracellular cytokine staining, cells were stimulated with 50 ng/ml PMA, 1 μg/ml ionomycin and 2.5 mg/ml brefeldin A (Sigma-Aldrich, St Louis, MO) in complete 1640 medium with 10% fetal calf serum (FCS) for 3 h and were washed once with ice-cold FACS staining buffer to remove the stimuli. The Cytofix/Cytoperm Kit (55414, BD Biosciences) was used for fixation and permeabilization in intracellular cytokine staining. All data were analyzed with FlowJo software (Tree Star, San Carlos, CA).

Fluorescence-conjugated antibodies against IFN-γ (XMG1.2), Vβ3 (KJ25), Vβ5.1/5.2 (MR-9.4), I-A (AF6), CD80 (16-10A1), and CCR6 (R6H1) were purchased from BD Biosciences. FITC mouse anti-human Ki-67 set (556026) was also purchased from BD Biosciences. The F4/80 (BM8) antibody was obtained from Biolegend (San Diego, CA). Antibodies against CD3e (2C11), CD4 (RM4-5), CD8α (53-6.7), CD11b (M1/70), CD25 (PC61.5), CD86 (24F), Foxp3 (FJK-16S), and TCRβ (H57-597) were from eBioscience.

### ELISA Analysis of cytokine expression

Bone-marrow-derived macrophages (BMMs) were generated as described previously^31^. Briefly, bone marrow was harvested from the femurs and tibias of mice and cultured in 1640 medium (GIBCO, Grand Island, NY) containing 10% FCS and 10% culture supernatant from M-CSF-expressing L929 cells for 6 d and then stimulated with different doses of LPS (Invivogen, Toulouse, France) for the indicated times. Thymus tissues were freezed in liquid nitrogen and lysate was prepared in the ELISA diluent buffere (00-4202-43, eBioscience) that was supplemented with 1 mM PMSF. IL-6 and TNF-α expression levels were measured in culture supernatants and thymus tissues using mouse ELISA Ready-SET-Go kits (88-7324-42 and 88-7064-22, eBioscience) according to the manufacturer’s protocols.

### Immunohistochemical staining

Thymus tissues were fixed in 4% buffered paraformaldehyde and embedded in paraffin. Sections were stained with hematoxylin and eosin, and IL-6 was detected using mouse IL-6 Immunohistochemical Kit (Im-20012, IBL-America, China)

### Western blotting

BMMs were stimulated with 100 ng/ml LPS or 100 μg/ml zymosan (Invivogen) for 0 to 120 min. Cells lysates were prepared on ice with lysis buffer (50 mM Tris, pH 7.4, 150 mM NaCl, 1% Triton X-100, 1 mM EDTA, pH 8.0) supplemented with protease inhibitor complete mini (Roche, Mannheim, Germany), as well as 1 mM PMSF, 1 mM Na_3_VO_4_, and 1 mM NaF (Sangon, Shanghai, China). After being incubated on ice for 20 min, the cell extracts were cleared by centrifugation at 13.2 k rpm for 15 min. Cell lysates were diluted with 2×SDS loading buffer, resolved via SDS-PAGE and transferred onto PVDF membranes (Millipore, Bedford, MA). Nonspecific binding was blocked with 5% nonfat milk in TBST buffer, and membranes were then incubated with primary antibodies for 2 h at room temperature, followed by 3 washes for 10 min each with TBST buffer. The blots were probed with antibodies against the indicated kinases. Antibodies against p-IκBα (Ser32), p-p38 (Thr180/Tyr182), p-JNK (Thr183/Tyr185), JNK, p-38 (Ser396), IRF3, and p-IKKα/β (Ser176/180) were purchased from Cell Signaling Technology (Beverly, MA). Antibodies against IκBα, ERK1/2, p-ERK and p38 (SC-728) were purchased from Santa Cruz Biotechnology (Santa Cruz, CA). Anti-p-BTK (Y223) and anti-BTK antibodies were purchased from Immunoway (Newark, DE). Bands were detected using HRP-conjugated secondary antibodies from Jackson ImmunoResearch Laboratories, Inc. (West Grove, PA) and developed using an enhanced chemiluminescence detection system (Perkin Elmer Life Sciences, Boston, MA) according to the manufacturer’s instructions. GAPDH was also detected with anti-GAPDH (Bioworld, Atlanta, GA) to ensure equal loading of proteins.

### Quantitative PCR

Total RNA was extracted from thymus tissues with TRIzol Reagent (Life Technologies, Grand Island, NY), and cDNA was synthesized from 1 μg of total RNA using the FastQuant RT kit (Tiangen, Beijing, China) according to the manufacturer’s instructions. Quantitative PCR was carried out with the SuperReal preMix plus (SYBR Green I) kit (Tiangen) and was performed on an ABI Prism 7900HT (Applied Biosystems, Foster City, CA). The primers used for quantitative RT-PCR amplification were as follows: mCCL20 forward/reverse 5′-TTGCTTTGGCATGGGTACTG-3′/5′-TCGTGTTGCTTGCTGCTTCTG-3′, mIL-1β forward/reverse 5′-ACAAGAGCTTCAGGCAGGCAGTA-3′/5′-ATATGGGTCCGACAGCACGAG-3′, mIL-2 forward/reverse 5′-TCCAGAACATGCAGAG-3′/ 5′-CCTGAGCAGGATGGAGAATTACA-3′, mIL-4 forward/reverse 5-′ CCCCGTGCTTGAAGAACAATTC-3′/5-′GGACTCATTCCCAGTACAGCTTTTC-3′, mIL-6 forward/reverse 5′-GAAGGAGGAGCTTGACCTTGG-3′/5′-AACCATGCCTATCATCCTTTGG-3′, mIL-10 forward/reverse 5′-ACCTGCTCCACTGCCTTGCT-3′/5′-GGTTGCCAAGCCTTATCGGA-3′, mTGF-β forward/reverse 5′-GGTTCATGTCATGGATGGTGC-3′/5′-TGACGTCACTGGAGTTGTACGG-3′, mTNF-α forwards/reverse 5′-TGGGAGTAGACAAGGTACAACCC-3′/ 5′-CATCTTCTCAAAATTCGAGTGACAA-3′ and mGAPDH forward/reverse 5′-GAGCCAAACGGGTCATCATCT-3′/5′-GAGGGGCCATCCACAGTCTT-3′. Transcript expression was normalized to GAPDH expression.

### Statistical analysis

Statistical analyses were performed using GraphPad Prism 5 software (Graphpad software, San Diego, CA). *P* values were calculated using unpaired Student’s *t* tests and one-way ANOVA. *P* < 0.05 was considered statistically significant.

## Additional Information

**How to cite this article**: Yang, C. *et al.* Non-classical MHC I-E negatively regulates macrophage activation and Th17 cell development in NOD mice. *Sci. Rep.* **5**, 12941; doi: 10.1038/srep12941 (2015).

## Supplementary Material

Supplementary Information
