# Supplementary material for: Non-classical MHC I-E negatively regulates macrophage activation and Th17 cell development in NOD mice
Source: Sci Rep. 2015 Aug 7;5:12941. doi: 10.1038/srep12941 (PMC4528198; doi:10.1038/srep12941)
Supplement: Supplementary Information [file srep12941-s1.pdf]

Supplementary data

Non-classical MHC I-E negatively regulates macrophage activation and Th17 cell development in NOD mice

Chunhui Yang*, Nining Guo*, Jinhua Liu*, Juhao Yang, Kai Zhu, Hui Xiao, Qibin Leng

Key Laboratory of Molecular Virology and Immunology, Institut Pasteur of Shanghai, Chinese Academy of Sciences, 320 Yueyang Road, Shanghai, China

* These authors contributed equally to this work.

Correspondence and requests for materials should be addressed to QL (qbleng@sibs.ac.cn) or HX (huixiao@sibs.ac.cn).

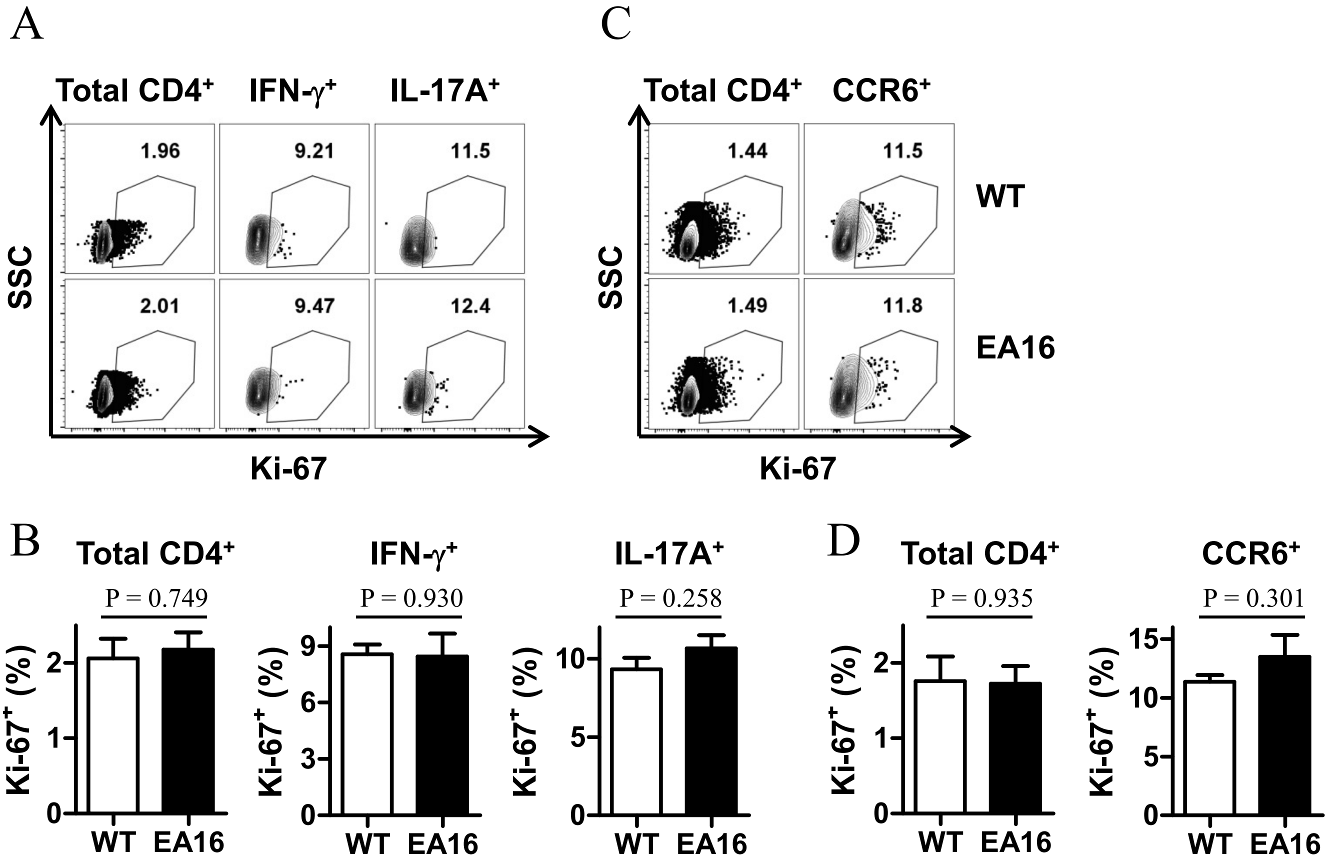

Fig. S1 Proliferation rate of Th17 cells from EA16 and WT NOD mice.

(A, B) The thymocytes from EA16 and WT NOD mice were stimulated with PMA/ionomycin for 3h as described previously (Fig. 1B-C), and intracellular IFN-γ and IL-17A expression were detected by flow cytometry. Percentages of Ki-67^{+} population in total $CD4^{+}$, IFN-$\gamma^{+}CD4^{+}$ or IL-$17A^{+}CD4^{+}$ cells were shown. (C, D) Cells from PDLNs from EA16 and WT NOD mice were stained with antibodies against CD4, CD8, CD44, CCR6 and Ki-67. Percentages of Ki-67^{+} population in total $CD4^{+}$ or $CD4^{+}CD44^{+}CCR6^{+}$ cells were shown. Data (mean ± SEM) are representative of three (A and B) or two (C and D) independent experiments with similar results. (n = 3 per genotype).

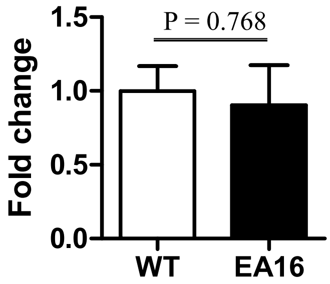

Fig. S2 CCL20 expression level in thymus tissue from EA16 and WT NOD mice. RT-PCR analysis of CCL20 expression in the thymi from EA16 (n = 6) and WT (n = 6) NOD mice.

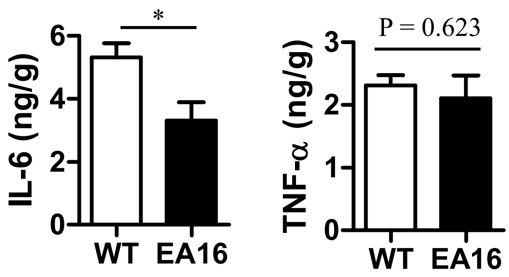

Fig. S3 Protein levels of IL-6 and TNF-α in thymus tissues from EA16 and WT NOD mice. Expression levels of IL-6 and TNF-α in thymus tissues from EA16 (n=6) and WT (n=6) NOD mice were analyzed with ELISA assay. *, ***P* < 0.05.**

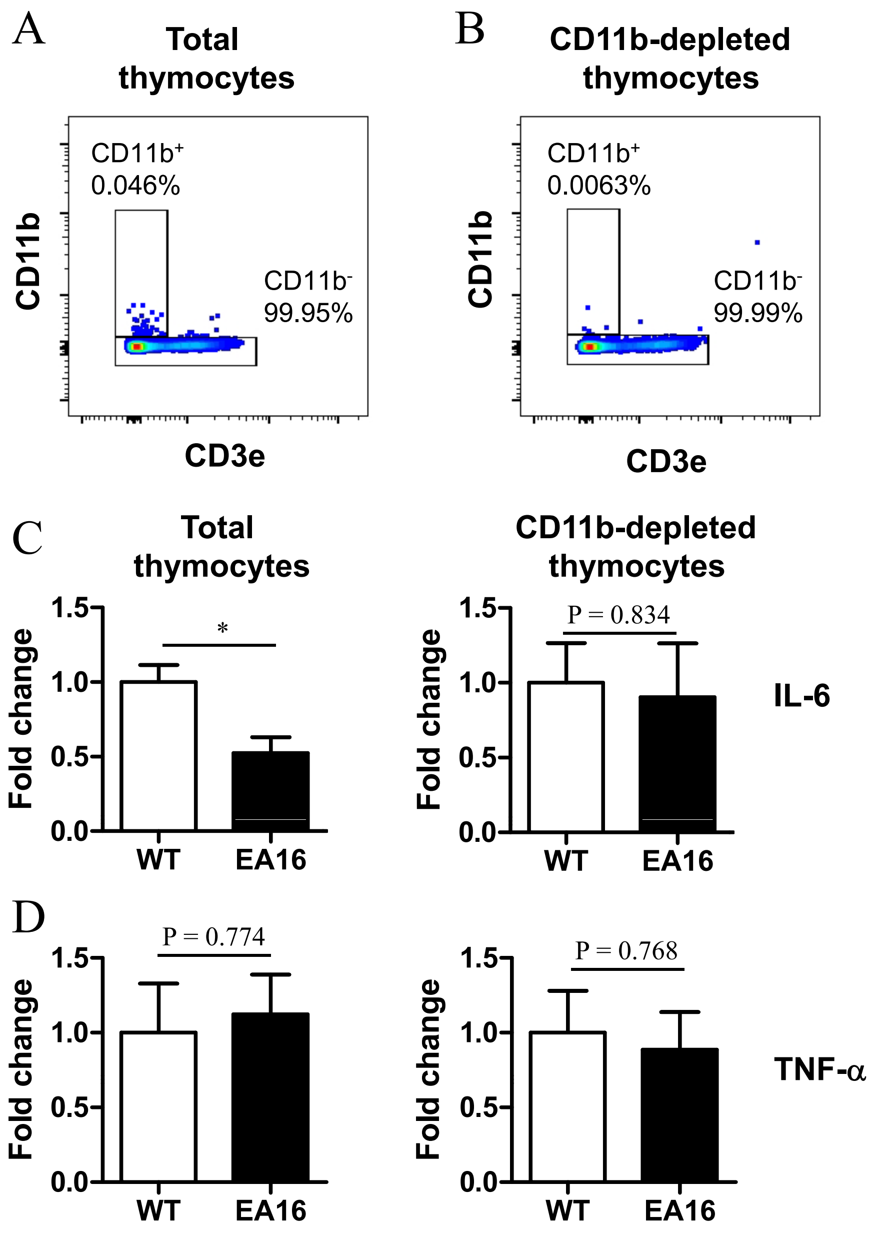

Fig. S4 IL-6 and TNF-α expression levels in different cell populations in thymus from EA16 and WT NOD mice. (A, B) Thymocytes from WT NOD mice were stained with antibodies against CD11b and CD3e before and after depletion of $CD11b^+$ cells and analyzed with flow cytometry. (A) Percentage of $CD11b^+$ population in thymus before magnetic activated cell sorting (MACS). (B) Percentages of $CD11b^+$ and $CD11b^-$ cells after MACS depletion. (C and D) qRT-PCR analysis of IL-6 (C) and TNF-α (D) expression in total thymocytes and CD11b-depleted population from EA16 (n=6) and WT (n=6) NOD mice. *, $P < 0.05$.

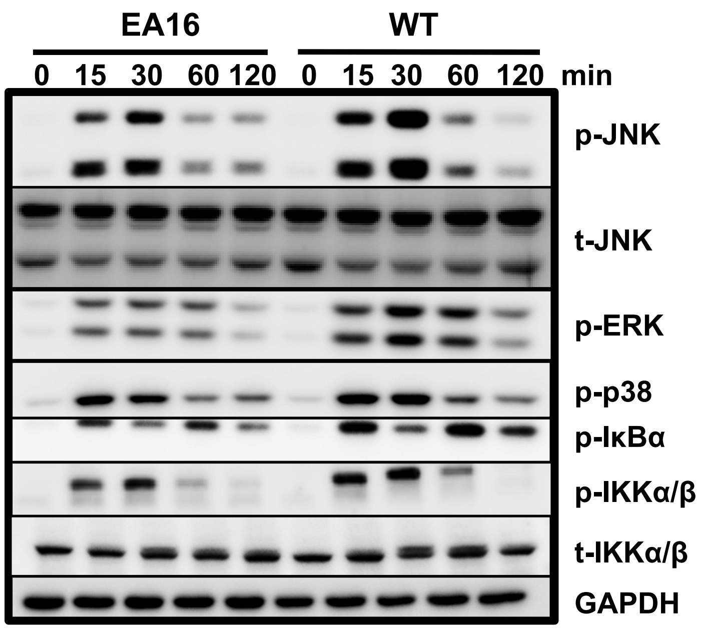

Fig. S5 I-E molecule attenuates zymosan-induced signaling in BMM cells. Immunoblot of phosphorylated (p-) or total (t-) proteins in lysates of WT or EA16 BMMs stimulated with zymosan (100 μg/ml) for the indicated times.
